# The Role of the VEGF Family in Atherosclerosis Development and Its Potential as Treatment Targets

**DOI:** 10.3390/ijms23020931

**Published:** 2022-01-15

**Authors:** Siarhei A. Dabravolski, Victoria A. Khotina, Andrey V. Omelchenko, Vladislav A. Kalmykov, Alexander N. Orekhov

**Affiliations:** 1 Department of Clinical Diagnostics, Vitebsk State Academy of Veterinary Medicine [UO VGAVM], 7/11 Dovatora Street, 210026 Vitebsk, Belarus; 2 Laboratory of Cellular and Molecular Pathology of Cardiovascular System, AP Avtsyn Research Institute of Human Morphology, 3 Tsyurupa Street, 117418 Moscow, Russia; nafany905@gmail.com; 3 Laboratory of Angiopathology, The Institute of General Pathology and Pathophysiology, 8 Baltiyskaya Street, 125315 Moscow, Russia; xxor2011@gmail.com; 4 Institute for Atherosclerosis Research, Osennyaya Street 4-1-207, 121609 Moscow, Russia; omi@bk.ru (A.V.O.); a.h.opexob@gmail.com (A.N.O.); 5 AP Avtsyn Research Institute of Human Morphology, 3 Tsyurupa Street, 117418 Moscow, Russia

**Keywords:** atherosclerosis, angiogenesis, inflammation, VEGF, cardiovascular diseases

## Abstract

The vascular endothelial growth factor (VEGF) family, the crucial regulator of angiogenesis, lymphangiogenesis, lipid metabolism and inflammation, is involved in the development of atherosclerosis and further CVDs (cardiovascular diseases). This review discusses the general regulation and functions of VEGFs, their role in lipid metabolism and atherosclerosis development and progression. These functions present the great potential of applying the VEGF family as a target in the treatment of atherosclerosis and related CVDs. In addition, we discuss several modern anti-atherosclerosis VEGFs-targeted experimental procedures, drugs and natural compounds, which could significantly improve the efficiency of atherosclerosis and related CVDs’ treatment.

## 1. Introduction

The VEGF is a family of heparin-binding proteins involved in angiogenesis, lymphopoiesis and lymphangiogenesis, resisting oxidative stress, regulation of lipid metabolism and inflammation. Originally, VEGF was discovered in 1983 as a tumour-secreted factor, causing disruption of intercellular contacts and enhancing vessels permeability [1]. The VEGF family consists of 5 gene products in humans, 3 of which regulate blood vessel growth (VEGF-A, VEGF-B and PlGF (placental growth factor), and 2 modulate lymphangiogenesis (VEGF-C and VEGF-D). Other known members include VEGF-E (virus source), VEGF-F (snake venom source) and EG-VEGF (endocrine gland-derived vascular endothelial growth factor). In addition, there are 3 VEGF receptors, VEGFR1 (Vascular Endothelial Growth Factor Receptor 1—*FLT1* gene) and VEGFR2 (*KDR* gene), mainly expressed in VECs (vascular endothelial cell), and VEGFR3 (*FLT4* gene), which is primarily expressed in LECs (lymphatic endothelial cells) [2,3]. With the regulation of lipid metabolism through the involvement of the lymphatic system in one-way absorption and transport of lipids from the gastrointestinal tract to the venous system [4].

Similarly, the VEGF family can also inhibit the inflammatory response, promote dilation and proliferation of lymphatic vessels and reduce oxidative stress, thus, prevent atherosclerosis progress [5]. Also, VEGF family genes are up regulated in many tumours, and their expression is correlated with the tumour stage. Thus, VEGF-targeted drugs (anti-angiogenic therapy) are a pioneering approach to the target tumour microenvironment and creates intratumor hypoxia, which leads to the selection of more invasive and resistant anti-angiogenic therapy cancer cells. However, hypoxic conditions up-regulate VEGF expression via *HIF-1α* (Hypoxia Inducible Factor 1 Subunit Alpha) transcription factor, thus, suggesting that blocking of VEGF-activating signalling mechanisms is necessary for effective anti-angiogenic therapy [6,7].

Additionally, VEGF signalling is involved in the regulation of ECs survival and adaptation to ER-stress via activation of UPR^ER^ (unfolded protein response) mediators through a Phospholipase C Gamma 1-mediated crosstalk with the mTORC1 (Mechanistic Target Of Rapamycin Kinase) complex without accumulation of unfolded proteins in the ER. Activation of Activating Transcription Factor 6 and PRKR-Like Endoplasmic Reticulum Kinase contributes to the survival effect of VEGF on ECs (endothelial cells) by positively regulating mTORC2-mediated phosphorylation of AKT (AKT Serine/Threonine Kinase 1) [8].

Further, we briefly discuss every member of the VEGF family, their general functions, regulation, role in lipid metabolism and specifically in atherosclerosis development and progression. However, the role of VEGF in cancer development and treatment is far beyond the focus of this review, and we wish to redirect interested readers to cited papers [6,7,9].

## 2. VEGFs Regulation and Functions in CVDs

### 2.1. VEGF-A

VEGF-A is the best-studied factor in the VEGF family. Acting via VEGFR-1 and VEGFR-2, VEGF-A mediates inflammation, angiogenesis and vascular permeability. VEGF-A is the core factor in the endothelial cells functions, physiological angiogenesis (forming blood vessels during tissue re-vascularisation) and pathological angiogenesis (as a marker of ischemic diseases, inflammation and microvascular occlusion) [10]. There are eight exons in VEGF-A, which produce after alternative splicing subtypes of different lengths (VEGF121, VEGF165 and VEGF206). The difference in the length of some exons (mostly 6–7th where heparin and heparin sulfate proteoglycan are encoded) explains different biological properties of those subtypes, while competition between isoforms makes their relations extremely complex [11]. The primary sites of *VEGF-A* expression are in the cardiovascular system (ECs, angioblasts and pericytes). However, under certain conditions (such as inflammation and hypoxia), *VEGF-A* could be expressed by many other cell types (chondrocytes, tumour cells, several types of blood cells, keratinocytes and others) [12].

The current scheme suggests that in CVDs, cardiomyocytes and ECs are often influenced by inflammatory factors and hypoxia, which stimulate HIFs (primary HIF-1α) expression. HIFs cause up-regulation of different pro-angiogenic factors (including VEGFs) to promote angiogenesis, VECs proliferation, endothelium integrity, improve vascular permeability and function. Thus, VEGFs compensate for the effects of ischemia and hypoxia and protect the injured myocardium [12]. In addition to HIFs, several other VEGFs activators and HIF-independent pathways have been identified. For example, under hypoxic conditions, ECs could produce TNFα, which activates HIFs via NF-kB-dependent way, thus, forming the TNFα-NF-κB-HIF-VEGF signalling cascade in ECs [13]. Also, *VEGF* expression could be up-regulated under limited oxygen and nutrition supply via the primary regulator of mitochondrial function Peroxisome Proliferator-Activated Receptor Gamma Coactivator 1-Alpha and Estrogen Related Receptor Alpha nuclear receptor [14]. Similarly, the restriction of thioamino acid was shown to initiate *VEGF* expression and angiogenesis in skeletal muscle via General Control Nonderepressible 2/Activating Transcription Factor 4 amino acid starvation pathway, thus, independent of hypoxia or HIF-1α [15]. As shown on cardiac macrophages, ANXA1 (Annexin A1), a phospholipase A2 inhibitor with anti-inflammatory activity, could stimulate the release of VEGF-A, thus, inducing cardiac repair and angiogenesis [16]. 

The expression level of VEGF is closely associated with lipid levels. As described earlier TNFα-NF-κB-HIF-VEGF signalling cascade could be interrupted by LDL (low-density lipoprotein), which suppress TNF Receptor Superfamily Member 1A, a TNFα receptor, and prevent HIFs expression in ECs and decrease VEGF level [13]. Also, E2F Transcription Factor 1, cell cycle controlling TF, was shown to inhibit VEGF (in a p53-dependent way) and PIGF (in a p53-independent way), thus reducing cardiac regeneration and neovascularisation ability after injury [17]. Furthermore, as mentioned earlier, Annexin A1 could also act via STAT3 (Signal Transducer And Activator Of Transcription 3) signalling pathway, when down-regulated ANXA1 inhibits the activation of STAT3 and further suppress *VEGF* expression [18]. 

**Figure 1:**
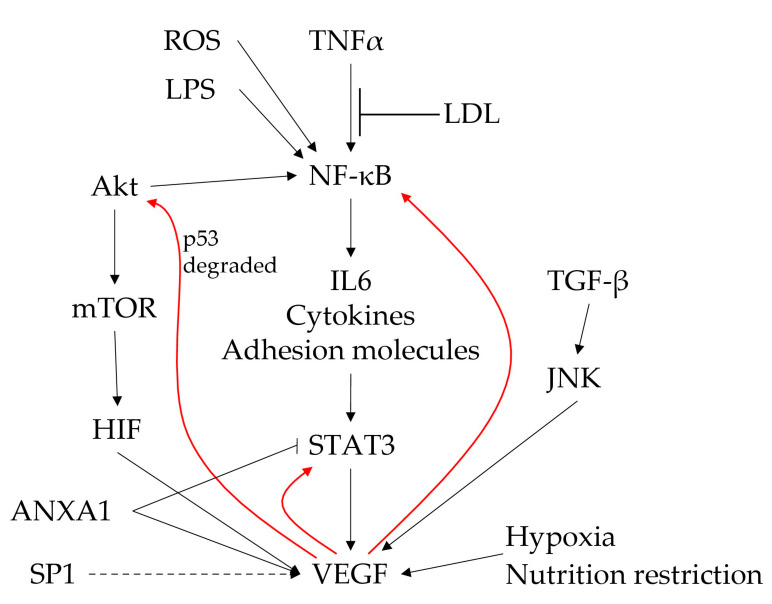
The central role of the VEGF and NF-κB pathway in the control of inflammation-mediated angiogenesis. Red arrows represent VEGF-mediated autofeedback loops.

The inflammation and angiogenesis processes are subjected to clear reciprocal modulation. NF-κB is one of the crucial factors involved in regulating inflammation, tumour suppression and control of proliferation. The NF-κB pathway is activated by multiple stimuli, including TNF-α, LPS and ROS. The activation of NF-κB can increase the release of IL-6 and, subsequently, activate STAT-3, leading to VEGF secretion. VEGF is linked to the inflammatory pathway at three critical points: 1) VEFG stimulates activation of STAT-3, creating an autofeedback loop to amplify its secretion; 2) VEGF also activates NF-κB; and 3) the Akt pathway, again supporting the feedback loop for its secretion via the IL-6/STAT-3 pathway (Figure 1). 

However, we wish to note that the relationship between inflammation and angiogenesis is an extremally complex topic. Tissue and organ microenvironment, different physiological and pathological conditions (such as disease, trauma, functional complication or cancer) could have various factors and pathways involved in the crosstalk between inflammation and angiogenesis. Therefore, we wish to redirect interested readers to the recent review [19]. 

### 2.2. VEGF-B

VEGF-B is a homologue of VEGF with two subtypes (the major one VEGF-B167, approximately 80% of the total VEGF-B, and minor one—VEGF-B186), able to bind only VEGFR-1. VEGF-B can bind cell surface or extracellular matrix elements and interact with NP-1 (neuropeptide-1). *VEGF-B* is expressed in many tissues and organs, mainly in the cardiovascular system (ECs, coronary artery smooth muscle cells, myocardium) and in the kidney, fat, lung and gallbladder [20]. Although VEGF-B is involved in the development of the cardiovascular system and the formation of embryonic myocardium, results from adult subjects suggest a role in the regulation of cardiac metabolism and cell survival, especially during pathological states such as heart failure and diabetic cardiomyopathy [21]. The discovered molecular mechanism relies on a bidirectional interaction between VEGF-B releasing cardiomyocytes and ECs. VEGF-B acts via ERK (also known as Mitogen-Activated Protein Kinase 1) to promote cell survival and regulate the expression of cell death genes [22]. 

There are several regulatory mechanisms known to regulate VEGF-B. Up-regulated via Akt/Sp1(Specificity Protein 1) pathway in ECs, VEGF-B acts synergistically with VEGF-A to induce VSMC (vascular smooth muscle cells) proliferation, ECs migration and to promote angiogenesis [20]. Also, VEGF-B is directly up-regulated by treatment with natural polyphenolic phytoalexin Resveratrol, which causes AMPK/eNOS (5′AMP-Activated Protein Kinase Catalytic Subunit Alpha-2/Nitric Oxide Synthase, Endothelial) pathway activation and increases NO (nitric oxide) production [23]. 

### 2.3. VEGF-C

VEGF-C and VEGF-D are secretory glycoproteins, sharing structural homology and primary functions. VEGF-C is the major factor regulating the physiological and pathological proliferation of lymphatic vessels. VEGF-C binds VEGFR-3 (the main receptor) on the surface of LECs; while the affinity towards VEGFR-2 is weaker, it also could promote angiogenesis. In addition, VEGF-C is involved in the repair of injured myocardium; thus, the levels of VEGF-C could serve as a marker in patients with suspected or confirmed CVDs [24,25]. *VEGF-C* is expressed in embryonic tissues (such as endothelium of large placental vessels, cells in maternal decidua and syncytiotrophoblasts) and many tissue and organs of adults (intestine, lymph nodes, heart, lung, brain and kidney) [26]. 

VEGF-C could be up-regulated by a high-salt diet, which accelerated left ventricular remodelling in a spontaneously hypertensive rat model system [27]. Because accelerated angiogenesis and lymphangiogenesis could be beneficial for tumour development, VEGF-C suppression was studied as a tool to inhibit tumour growth. For example, miR-182-5p directly down-regulates VEGF-C and inhibits tumorigenesis, lymphangiogenesis and angiogenesis of colon cancer [28]. Also, several anti-cancer drugs (such as kolaviron and norcantharidin) were shown to down-regulate or suppress VEGF-C [29,30]. 

### 2.4. VEGF-D

The VEGF-D is a secondary to VEGF-C lymphangiogenesis factor, compensating VEGF-C deficiency. In comparison to VEGF-C, VEGF-D has higher angiogenic potential. The high level of VEGF-D was associated with several CVDs (atrial fibrillation, ischemic stroke and coronary artery disease) [31,32]. *VEGF-D* is highly expressed in the embryonic lungs, while in adulthood also in other organs (small intestine, lung and heart) [33]. 

The molecular mechanisms that regulate *VEGF-D* expression are not fully understood. Inflammation can induce VEGF-D/VEGFR-3 signalling pathway and subsequent lymphangiogenesis. In particular, proinflammatory cytokines activate the NF-kB pathway and promote *Prox1* (Prospero Homeobox 1) and *VEGFR-3* expression, resulting in enhanced production and response of VEGF-A, VEGF-C and VEGF-D, thus, further promoting lymphangiogenesis [34]. TGF-β1 (transforming growth factor-β1) in fibroblasts could down-regulated VEGF-D acting via the Jun NH2-terminal kinase signalling pathway [35]. Suppression of all types of VEGFs was shown after lipopolysaccharide treatment (inflammation mimicking). Also, *VEGF-D* expression could be regulated on mRNA level [reviewed in [36]].

**Figure 2:**
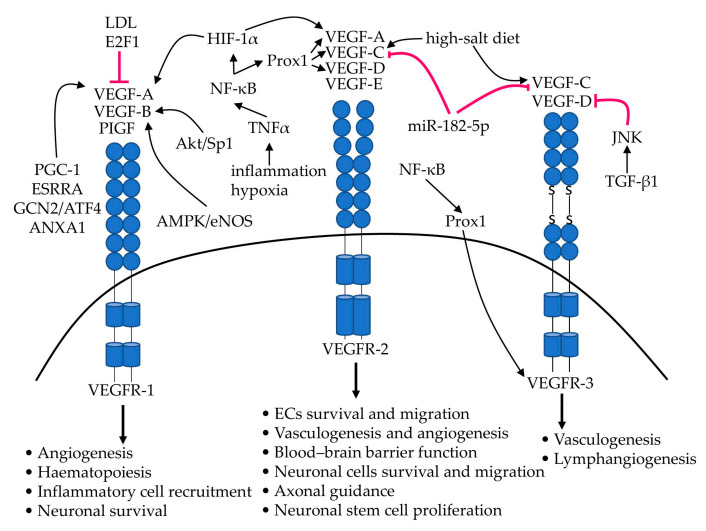
The VEGF family of growth factors. The main VEGF isoforms (VEGF-A, VEGF-B, VEGF-C, VEGF-D and PlGF) and their target the receptor tyrosine kinases (VEGFR-1, VEGFR-2 and VEGFR-3). The key regulatory pathways described in the text and significant effects on receptor-expressing cell types are indicated.

The main molecular mechanisms discussed in this section, involved in the regulation of the VEGF family expression and significant effects on receptor-expressing cell types, are summarised in Figure 2. However, the VEGF/VEGFR’s regulation is a complex multifaceted signalling web, with many VEGF/VEGFR-interacting proteins (such as protein tyrosine phosphatases, neuropilins, integrins and proteoglycans) and other endothelial signalling cascades involved. Therefore, we wish to redirect interested readers to the following excellent review [37].

In total, different VEGF family members and isoforms of individual VEGF’s could represent an overall course of CVDs and patient’s health conditions, provide valuable information for prognosis and promising targets for treatment. 

## 3. The Role of VEGFs in Lipid Metabolism

### 3.1. VEGF-A

VEGF-A is a part of the Nrp1/VEGFR-1 signalling pathway, which is involved in the regulation of CM (chylomicrons) absorption in intestinal lymphatic vessels. NRP1 (Neuropilin 1) is a receptor, participating in different types of signalling pathways that control cell migration. VEGF-A/Nrp1/VEGFR-1 signalling pathway regulates chylomicrons entering the chylous duct; thus, dysregulation could cause inhibition of CM absorption [4]. Also, VEGF-A decreases the activity of LPL (plasma lipoprotein lipase), resulting in the accumulation of triglycerides in large lipoprotein granules, including chylomicrons and very low-density lipoprotein, which resulted in the change of atherosclerosis promotion [38]. Simultaneously, the blood levels of lipids could regulate *VEGF-A* expression and affect its biological activity—a high-fat diet leads to the increased serum level of VEGF-A [39]. 

### 3.2. VEGF-B

VEGF-B is known for its lipid-lowering effect. Acting via VEGFR-1/AMPK and NP-1, VEGF-B regulates transcription of vascular fatty acid transporters; thus, it controls the uptake of fatty acids from the circulating lipids by ECs, their further transcytosis to lower tissues and closely related lipid utilisation of mitochondria [40]. In addition, VEGF-B regulates cholesterol level and glucose uptake via LDLR-mediated cholesterol uptake, where enhancement of VEGF-B resulted in lower plasma membrane cholesterol load [41]. However, VEGF-B inhibition in type 2 diabetes patients was shown to prevent lipid accumulation, improve glucose tolerance and insulin sensitivity, and reduce the content of islet TG (triglyceride) [42]. Also, a lipid-lowering function of VEGF-B was shown in the muscle and liver of mice on HFD, where VEGF-B induces the expression of carnitine palmitoyltransferase-1, Acetyl-CoA Carboxylase 1 and AMPK, thus, participating in de novo fatty acid biosynthesis and mitochondrial fatty acid beta-oxidation [43].

### 3.3. VEGF-C

The VEGF-C/VEGFR-3 signalling pathway is involved in the regulation of the lipid transport of intestinal lymphatic vessels, where lipids are absorbed from food. The blockage of VEGFR-3 signalling resulted in dysregulated TG absorption to the lymphatic system, resulting in TGs retention in the intestinal epithelial cells, reduced levels of TGs in plasma and increased excretion of TGs and free fatty acids into faeces [44]. Systemic blockade of VEGFR-3 caused the reduction of macrophages infiltration in adipose tissue, lower levels of lipids accumulated in the liver and improved insulin sensitivity, thus, suggesting VEGF-C/VEGFR-3 as a promising target in treatments of insulin resistance-based diseases (obesity, diabetes and metabolic syndrome) [45]. Similarly, VEGF-C-deficient mice had impaired lipid absorption, increased faecal levels of fatty acids and cholesterol, and improved glucose metabolism and resistance to obesity when fed on HFD [46]. Those effects were confirmed on mice overexpressing VEGF-C, which resulted in insulin resistance, weight gain and progress of metabolic syndrome [47]. 

### 3.4. VEGF-D

Similar to VEGF-C, VEGF-D plays an essential role in the regulation of lipid metabolism. Down-regulation of the VEGF-D/VEGFR-3 signalling pathway resulted in the dysregulation of lipid biosynthesis and peroxisome β-oxidation pathways. VEGF-D knockout (VEGF-D^−/−^LDLR^−/−^ApoB^100/100^ mice) leads to decreased VECs penetration for large chylomicrons and increased TGs and cholesterol plasma levels [48]. Adipose tissue-specific VEGF-D overexpression in the HFD mice model resulted in de novo lymphangiogenesis, enhanced glucose clearance, lower insulin levels, and reduced liver triglycerides. Those results suggest a new potential target in the treatment of the metabolic syndrome associated with obesity [49]. 

The VEGF family is closely tied to the lymphatic system, which further regulates lipid metabolism, inflammation and redox potential. However, in clinical practice, lymph examination is seldom used to diagnose or monitor CVDs progression. Discussed papers suggest that evaluation of VEGFs in lymph could be helpful for diagnosis and as a target for CVDs treatment. Further, we discuss the role of the VEGFs/VEGFRs signalling pathways in atherosclerosis. 

## 4. Atherosclerosis

Atherosclerosis is a disease characterised as chronic non-resolved inflammation and cholesterol accumulation in the vascular wall of the medium and large arteries [50]. In atherosclerosis, neovascularisation is involved in unstable plaque formation and the risk of rupture [51]. Coronary artery disease and atherothrombosis are two critical pathological processes resulting from atherosclerosis. CAD (coronary artery disease) pathology is caused by atherosclerosis and is characterised by constricted or blocked coronary arteries. Angiogenesis mediates the growth and vulnerability of plaques and promotes the influx of erythrocytes and inflammatory cells, leading to the plaque break and worsening CAD condition [52]. Consequently, CAD patients have increased serum and plasma levels of VEGF-A and IL-18, which could be used as a marker for revascularisation and identification of CAD severity [53]. As a complex inflammatory pathological process, atherothrombosis is characterised by lipid deposition in the arterial wall, recruitment, and accumulation of circulating leukocytes, leading to plaque formation and growth. Further, unstable plaque could break and trigger thrombus formation [54].

**Figure 3:**
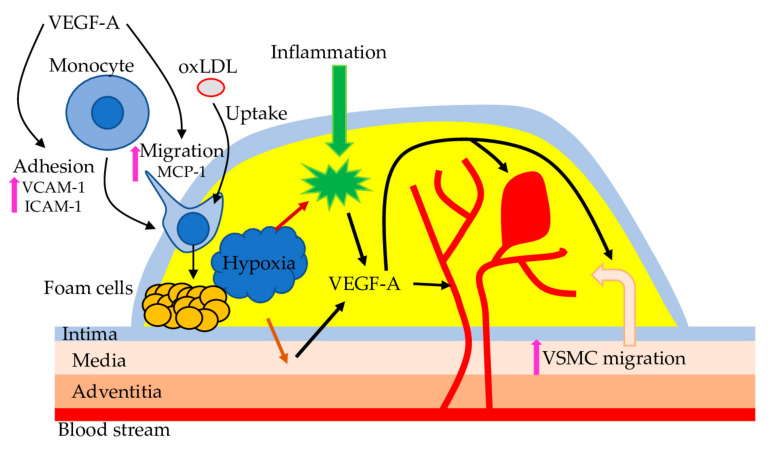
VEGF-A and atherosclerosis. Hypoxia and inflammation in the atherosclerotic plaque trigger *VEGF-A* expression in VSMC and macrophages. VEGF-A promotes endothelial expression of adhesion molecules and secretion of chemotactic substances to stimulate monocytes’ recruitment, adhesion, and transmigration into the blood vessel wall. Further, monocytes differentiate into macrophages and accumulate oxidized LDL (oxLDL), leading to foam cell formation. In addition, VEGF-A promotes migration of VSMC from the tunica media of the blood vessel wall into the plaque, which could initiate platelet activation and aggregation, resulting in thrombus formation. Also, VEGF-A acts on the blood vessel to promote angiogenesis, associated with haemorrhage.

VEGF-A is the best-studied VEGF family member and, based on the rabbit’s experiments, is identified as a marker of atherosclerosis [55]. VEGF-A has several beneficial and harmful functions related to atherosclerosis. For example, VEGF-A protects ECs by stimulating the expression of anti-apoptotic proteins and NO synthesis [56]. However, VEGF-A also prevents repair of the endothelial lesion that can induce atherogenesis, promotes monocyte adhesion, transendothelial migration and activation, improves the expression of monocyte chemoattractant protein-1 and adhesion protein, endothelial permeability. Also, the application of anti-angiogenic factors effectively reduces atherosclerosis development and progression in different models [57]. In atherosclerosis, carotid intima-media thickness is negatively correlated with levels of VEGF-A and ET-1 and positively—with NO level; thus, VEGF-A could serve as a marker to monitor atherosclerosis and related pathologies [58]. However, a recent study on Caucasian patients with early-onset CAD suggests that the level of circulating VEGF is only marginally associated with an increased risk for atherosclerosis [59]. Thus, the causative role of VEGF-A in atherosclerosis requires further investigation (Figure 3). 

**Figure 4:**
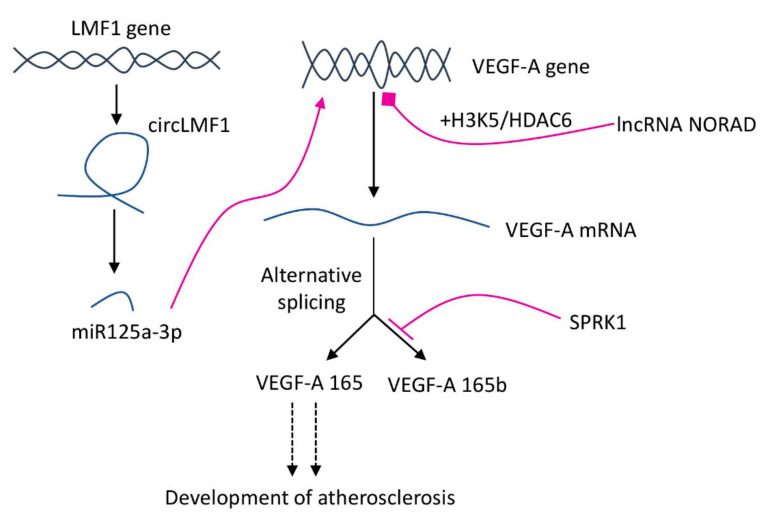
RNA-mediated regulation of *VEGF-A* expression and splicing isoforms balance. SPRK1 binds VEGF-A mRNA and shifts balance to the production of pro-angiogenic VEGF165. lncRNA NORAD (non-coding RNA activated by DNA damage) suppresses VEGF-A transcription with the help of H3K9 deacetylation and HDAC6 (Histone Deacetylase 6). Circular RNA originated from LMF1 (lipase maturation factor 1) interacts with miR-125a-3p, for which VEGF-A is a downstream target.

Recent research suggested that alternative splicing of VEGF-A play a crucial role in the pathogenesis of atherosclerosis. ApoE^−/−^ mice on HFD had more AECs (aortic endothelial cells), AEC proliferation and aortic infiltrated macrophages. Also, in AECs and macrophages derived from HFD mice, VEGF-A splicing isoforms were shifted to pro-angiogenic VEGF165 [60]. The endogenous balance of pro/anti-angiogenic VEGF-A splice isoforms is regulated by serine/arginine-rich splicing factor protein kinase 1, which binds to the VEGF-A mRNA and produce VEGF165 [61] (Figure 4). 

Also, lncRNA NORAD plays an essential role in vascular endothelial cell injury and atherosclerosis development. As shown on HFD fed ApoE^−/−^ mice and HUVECs, the expression of lncRNA *NORAD* was increased in ox-LDL-treated HUVECs and thoracic aorta of atherosclerotic mice. At the same time, the lncRNA NORAD knockdown alleviated vascular endothelial cell injury and atherosclerosis development in both models (in vitro and in vivo). In ox-LDL-treated HUVECs, VEGF gene transcription was suppressed by nucleus lncRNA NORAD enhancing H3K9 deacetylation and recruiting HDAC6 (Histone Deacetylase 6) to the VEGF gene promoter [62]. In addition to lncRNA, an ability to regulate VEGF-A was proven for circRNA (circular RNAs) originated from 6–8 exons of the LMF1 (lipase maturation factor 1). The molecular mechanism of the circLMF1 regulation of VEGF-A relies on the miR-125a-3p, for which VEGF-A is a direct downstream target. In the human aortic VSMCs atherosclerosis model, circLMF1 deficiency inhibited cell viability, cell cycle progression, and migration [63]. These results suggest an essential role of the RNA-mediated mechanisms in the pathogenesis of atherosclerosis and new potential targets for pharmaceutical intervention. 

## 5. VEGF as a Therapeutic Target

Nowadays, angiogenesis inhibitors are widely used to prevent the formation of new blood vessels in tumours, which results in interruption of tumours growth and cause tumour regression. In 2004 Bevacizumab was the first approved drug targeted against all isoforms of VEGF-A; since that, similar several medicines have been developed and successfully used for the treatment of many types of cancer (colorectal cancer, non-squamous cell lung cancer, metastatic renal cell carcinoma and others) reviewed in [64,65]. Application of VSP (VEGF signalling pathway) inhibitors cause an anti-angiogenic effect by influencing the survival and proliferation of ECs and VSMCs. There are two basic pharmacological mechanisms of the VSP inhibitors: monoclonal antibodies against the extracellular VSP components and inhibition of the intracellular domain of the tyrosine kinase (VEGFRs). The second mechanism is not specific because tyrosine kinases are used by many signalling systems [66]. Not surprisingly, such drugs have significant cardiovascular toxicities, which are not surprising due to the crucial role of VEGF in the development and functions of the vasculature and heart. Collectively, VSP inhibitors increase the odds of atherosclerosis, hypertension, arterial thromboembolism, cardiac ischemia and cardiac dysfunction, heart failure. Thus, it is crucial to evaluate the risk-benefit balance of VSP inhibitors’ application because these agents are primarily used in patients with metastatic malignancy, where the range of available treatments is limited [67]. Further in this section, we discuss recent research in VSP-targeted anti-atherosclerosis treatment. 

### 5.1. Experimental Procedures

VEGF is the powerful pro-angiogenic factor promoting re-endothelialisation and ameliorating neointima formation after vascular injury. However, VEGF application in clinical practice requires an effective targeted delivery system because short half-life, poor biostability in vivo and several overdose-related adverse effects (such as escalated inflammation and trigger neoplasms development) significantly limit VEGF usage [68]. Macrophages can reach sites of inflammation, injury and tumours; thus, they could be used as a carrier of drugs or genes to those specific sites [69]. Application of VEGF-modified macrophages therapy on atherosclerosis-prone mice with wire-induced carotid artery injury model resulted in accelerated re-endothelialisation and attenuated neointima formation. Also, the level of VEGF protein in tissues of injured arteries was increased, and NO production in the culture medium of VEGF-modified macrophages was enhanced. Those results suggest that VEGF-modified macrophages could be used to repair injured arteries and sustain local NO production and VEGF secretion [70]. 

Restenosis is a common adverse event of endovascular procedures used to treat vascular damage from atherosclerosis and other operations. Restenosis is the recurrence of stenosis (a narrowing of a blood vessel) leading to restricted blood flow. Currently, the most effective therapeutic option to prevent restenosis is a complete re-endothelialisation followed by inhibition of smooth muscle cell proliferation [71]. Application of bilayered nanoparticles was shown to sequentially release VEGF plasmids from the outer layer and paclitaxel from the core to promote endothelial regeneration and prevent restenosis. Administration of such nanoparticles to the injured aortic wall of a rabbit model of atherosclerosis resulted in an increased level of VEGF and decreased level of C-reactive protein (CRP), followed by a rapid re-endothelisation and inhibition of restenosis, thus, ameliorating atherosclerosis [72]. 

Atherosclerosis is one of the most common complications in chronic kidney disease and end-stage renal failure patients under maintenance haemodialysis. MicroRNA-126 is an endothelial-cell-specific miRNA involved in the regulation of vascular integrity, angiogenesis and also the development and progression of atherosclerosis [73]. A recent study suggested using miR-126 and VEGF serum levels as a biomarker to monitor atherosclerosis progression in patients under haemodialysis. Obtained results showed a negative correlation of miR-126 with intima-media thickness and plaque area, but a positive association between VEGF and intima-media thickness and plaque area. [74,75]. Mechanically, the anti-atherosclerotic effect of blood purification relies on the improved lipid metabolism (decreasing TG and enhancing the concentration of HDL types), thus lowering the occurrence of atherosclerosis in patients under haemodialysis [76].

The lymphatic system is a crucial contributor to the development and progression of atherosclerosis [77]. VEGF-C/VEGFR-3 signalling is important for the normal development of lymphatic vessels, the process of lymphangiogenesis and stimulation of lymphatic pumping [44]. However, recent experiments also suggested that VEGF-C/VEGFR-3 be used as a target for preventing and treating atherosclerosis. VEGF-C 152S (analogue to the human VEGF-C 156S mutant and a selective VEGFR-3 agonist) was injected in Ldlr^−/−^ mice fed on HFD, resulting in improved outcomes lymphatic molecular transport and inflammatory cell migration through the lymphatics, limited plaque formation and macrophage accumulation. Also, the contraction frequency of the collecting lymphatic vessels was significantly increased and resulted in enhanced plaque stabilisation. The expression levels of VEGFR3 and Forkhead Box C2 on lymphatic ECs were upregulated [5].

Similarly, plaque stabilisation was achieved with F8-antibody/VEGF-C conjugates applied on ApoE^−/−^ mice atherosclerosis model [78]. Furthermore, promising NO-releasing drug MPC-1011 used in the rat hindlimb ischemia model of PAD (peripheral arterial disease) was shown to act in the NO/cGMP/VEGF-dependent way [79]. PAD is caused mainly by atherosclerosis and is characterised by impaired blood flow to the lower extremities, severe exercise intolerance and claudication pain [80]. MPC-1011 treatment improved vascular remodelling and stimulated the release of VEGF, SDF1 (Stromal Cell-Derived Factor 1) and increased tissue cGMP levels, potentiated proliferation and migration of ECs, reduced platelet activation and aggregation [79]. 

### 5.2. Natural Compounds

In addition to chemically-produced drugs, there are also several natural compounds with VEGF-mediated anti-atherosclerotic effects. For example, Tetramethylpyrazine (TMP) and Paeoniflorin (PF) (active ingredients of Ligusticum chuanxiong Hort. and Radix Paeoniae Rubra, respectively) both could alleviate atherosclerosis [81,82]. As it was shown on ox-LDL induced HUVECs angiogenesis, TMP and PF treatment (separately and in combination) inhibited *VEGF* and *VEGFR2* expression and decreased expression of angiogenesis-related factors Notch1 (*NOTCH1*), Jagged1 (*JAG1*), and Hes1 (*HES1*), which might contribute to the stability of plaques in atherosclerosis [83]. 

Resveratrol is a popular natural food supplementation that improves lifespan and affects any human disease [84]. Among others, anti-inflammatory and anti-atherogenic actions of resveratrol were shown to protect properties against atherosclerosis-associated endothelial dysfunction and senescence [85]. Furthermore, as was recently demonstrated on the rabbit model of atherosclerosis, oral supplementation of resveratrol has anti-inflammatory and anti-atherosclerotic effects, decreases serum levels of VEGF and CRP, and reduces atherosclerotic lesions’ formation and development [86]. 

Triptolide (TPL) is a natural compound isolated from the herb *Tripterygium wilfordii* Hook F. with many studied biomedical properties (anti-inflammatory, anti-cancer and anti-atherosclerotic) [87,88]. Further, miR-92a was defined as a crucial atherosclerosis regulator and biomarker [89,90]. Application of triptolide on in vitro HMEC-1 (human dermal microvascular endothelial cells) angiogenesis model resulted in miR-92a-mediated downregulation of the *eNOS*, *VEGFR2* and *VEGF* expression, thus, could potentially suppress atherosclerosis [91]. 

Recently it was shown that peonidin and petunidin, two anthocyanins naturally occurring in berries and grapes, regulate angiogenesis and atherogenesis. In particular, in TNF-α stimulated pro-inflammatory environment, peonidin and petunidin prevent monocyte adhesion to HUVECs, reduce VCAM-1, E-selecting and VEGF production [92]. Not surprisingly, many other natural compounds directly affect the expression of *E-selectin* and *VCAM-1*, two crucial adhesion molecules involved in the initiation of the atherosclerotic process [93]. One of the possible mechanisms of regulation of VEGF’s expression and signalling could rely on the direct hydrophobic interaction between VEGF and polyphenol molecules, causing a change in the secondary structure of the protein, thus, inhibiting further VEGFR2 signalling [94]. 

In total, discussed papers suggest VEGF proteins and VEGF/VEGFR signalling pathways as new targets for atherosclerosis treatment. Currently, there are several experimental medical procedures, new drugs and natural compounds acting on VSP. Also, VSP components could be used as novel biomarkers associated with CVDs. Discussed gene and protein therapies, transplantation and nanotechnology-based concepts could be helpful in both atherosclerosis treatment and a deeper understanding of CVDs molecular mechanisms. 

## 6. Conclusions

The VEGF proteins are critical regulators of angiogenesis, lymphangiogenesis, lipid metabolism and inflammation. In this review, we discussed the current knowledge on the regulation and functions of VEGFs, their role in lipid metabolism and atherosclerosis. Additionally, VEGFs have a high potential as biomarkers for prognosis, monitoring CVDs progression and severity. Further, scientific advances have led to the discovery of several VEGFs’ targeted experimental procedures for treating atherosclerosis and related CVDs (such as CAD, PAD and atherothrombosis). Also, we discussed several drugs and natural compounds that directly or indirectly affect VEGFs’ level of expression or signalling; thus, they could be used in anti-atherosclerosis therapy. While presented papers provide convincing evidence of the successful application of VEGF-targeted anti-atherosclerosis therapies and treatments, we have to note that majority of obtained results were conducted in animal model systems and in vitro. Thus, further detailed investigation of described approaches is required before it can be applied in real-life clinical practice. 

## Data Availability

Not applicable.

## Abbreviations

ACE2	angiotensin-converting enzyme 2
ADSC	adipose-derived stem cell
AECs	aortic endothelial cells
AMPK	5′AMP-Activated Protein Kinase Catalytic Subunit Alpha-2/l
CAD	coronary artery disease
CM	chylomicrons
CRP	C-reactive protein
CVDs	cardiovascular diseases
ECs	endothelial cells
eNOS	Nitric Oxide Synthase, Endothelia
HIF-1α	Hypoxia Inducible Factor 1 Subunit Alpha
LDL	low-density lipoprotein
LECs	lymphatic endothelial cells
LMF1	lipase maturation factor 1
LPL	plasma lipoprotein lipase
NO	nitric oxide
PAD	peripheral arterial disease
PF	Paeoniflorin
SDF1	Stromal Cell-Derived Factor 1
STAT3	Signal Transducer And Activator Of Transcription 3
TC	total cholesterol
TG	triglyceride
TGF-β1	transforming growth factor-β1
TMP	Tetramethylpyrazine
VCAM-1	Vascular Cell Adhesion Molecule 1
VECs	vascular endothelial cell
VEGF	vascular endothelial growth factor
VEGFR1	Vascular Endothelial Growth Factor Receptor
VSP	VEGF signalling pathway
